# Biosynthesis of Silver Nanoparticles on Orthodontic Elastomeric Modules: Evaluation of Mechanical and Antibacterial Properties

**DOI:** 10.3390/molecules22091407

**Published:** 2017-08-25

**Authors:** Alma E. Hernández-Gómora, Edith Lara-Carrillo, Julio B. Robles-Navarro, Rogelio J. Scougall-Vilchis, Susana Hernández-López, Carlo E. Medina-Solís, Raúl A. Morales-Luckie

**Affiliations:** 1 Facultad de Odontología, Universidad Autónoma del Estado de México, Jesús Carranza y Paseo Tollocan, 50120 Toluca, Estado de México, Mexico; alm_124@hotmail.com (A.E.H.-G.); jubarona13@yahoo.com.mx (J.B.R.-N.); 2 Centro de Investigación y Estudios Avanzados en Odontología, Universidad Autónoma del Estado de México, Jesús Carranza y Paseo Tollocan, 50130 Toluca, Estado de México, Mexico; rogelio_scougall@hotmail.com; 3 Facultad de Química, Universidad Autónoma del Estado de México, Paseo Colón intersección Paseo Tollocan S/N, 50120 Toluca, Estado de México, Mexico; shernandezlopez@yahoo.com; 4 Área Académica de Odontología, Instituto de Ciencias de la Salud, Universidad Autónoma del Estado de Hidalgo, 42039 Pachuca, Hidalgo, Mexico; cemedinas@yahoo.com; 5 Centro Conjunto de Investigación en Química Sustentable UAEM-UNAM, Universidad Autónoma del Estado de México, Carretera Toluca-Atlacomulco Km 14.5, San Cayetano, 50200 Toluca, Estado de México, Mexico

**Keywords:** biosynthesis, silver nanoparticles, orthodontic elastomeric modules, physical properties, antibacterial activity

## Abstract

In the present study, silver nanoparticles (AgNPs) were synthesized in situ on orthodontic elastomeric modules (OEM) using silver nitrate salts as metal-ion precursors and extract of the plant *Hetheroteca inuloides* (*H. inuloides*) as bioreductant via a simple and eco-friendly method. The synthesized AgNPs were characterized by UV-visible spectroscopy; scanning electron microscopy-energy-dispersive spectroscopy (SEM-EDS) and transmission electron microscopy (TEM). The surface plasmon resonance peak found at 472 nm confirmed the formation of AgNPs. SEM and TEM images reveal that the particles are quasi-spherical. The EDS analysis of the AgNPs confirmed the presence of elemental silver. The antibacterial properties of OEM with AgNPs were evaluated against the clinical isolates *Streptococcus mutans*, *Lactobacillus casei*, *Staphylococcus aureus* and *Escherichia coli* using agar diffusion tests. The physical properties were evaluated by a universal testing machine. OEM with AgNPs had shown inhibition halos for all microorganisms in comparison with OEM control. Physical properties increased with respect to the control group. The results suggest the potential of the material to combat dental biofilm and in turn decrease the incidence of demineralization in dental enamel, ensuring their performance in patients with orthodontic treatment.

## 1. Introduction

The presence of fixed appliances on tooth surfaces makes the teeth cleaning process difficult, favoring dental biofilm accumulation [1]. After the bonding of orthodontic appliances, there are documented increases in the amounts of *Streptococcus mutans* and *Lactobacilli* in the saliva and dental plaque of patients [2]. These microorganisms have been identified as the main pathogens in dental caries and their presence increases the risk for decalcification [3]. White spot lesion (WSL) around brackets is a major complication in patients with fixed orthodontic treatments, especially those with poor oral hygiene. These lesions are due to demineralization of enamel by acids from biofilms around the brackets [4,5]. Development of WSL during fixed appliances therapy can occur rapidly. Studies by O’Reilly et al. and Øgaard et al. showed development of clinically visible WSL in orthodontic patients that occurred in four weeks or less [6,7]. Gorelick et al. studied the incidence of WSL in orthodontic patients and found that almost 50% of orthodontic patients developed at least one WSL during the course of treatment [8,9,10].

The method of ligation of orthodontic arch wires is a relevant factor that accounts for dental biofilm retention. In the search for more practical and efficient orthodontic accessories, elastomeric modules (ligatures) have been suggested as the material of choice to connect stainless steel arch wires to brackets instead of metallic ligatures [11]. Orthodontic elastomeric modules (OEM) are synthetic elastics made of polyurethane material, with advantages such as quickness of application, patient comfort and less expensive than self-ligation clips [12]. Apart from its practical benefits, it is evident from the literature review that elastomeric ligatures exhibit a greater number of microorganisms in the plaque around the brackets when compared with steel ligatures [13].

Forsberg et al. evaluated the microbial colonization of twelve patients treated with fixed orthodontic appliances and reported that the lateral incisor attached to the arch wire with an elastomeric ligature exhibited a greater number of microorganisms in dental plaque. They also reported a significant increase in the number of *S. mutans* and *Lactobacilli* in saliva after the insertion of fixed appliances [14,15]. The rough surface and the absorption properties of elastomeric ligatures further contribute to the formation of bacterial plaque on their surfaces, resulting in accumulation of a higher number of microorganisms on tooth surfaces [16]. They recommended that the use of elastomeric ligatures should be avoided in patients with inadequate oral hygiene because elastomeric ligatures will significantly increase microbial accumulation on tooth surfaces adjacent to the brackets, leading to a predisposition for the development of dental caries and gingivitis [14].

Elastomers in oral cavity would rapidly become coated with salivary proteins and biofilm help to deterioration of their physical properties. If elastomeric modules lack adequate physical properties, clinical applications will be difficult and time-consuming. The latter may cause undesirable tooth movement and prolongs orthodontic treatment [12].

Plaque control is a critical factor that might limit that implantation and settling of causal microorganisms from caries and periodontal disease [17].

During orthodontic treatment, some preventive measures may be adopted to protect tooth structure. Oral hygiene instruction and supervision, nutritional counseling, plaque staining, professional tooth cleaning and daily mouth rinses with fluoride solution are some methods used by orthodontist that depend on the cooperation of the patient. Ideal prevention should not depend on patient cooperation [18,19].

More recently, these general measures are increasingly being supplemented with specific recommendations for the treatment of bracket problem zones. Some noteworthy methods include fluoride (F)-releasing adhesives and fluoride-releasing elastomeric ligatures ties [19,20]. Nevertheless, the protocols of fluoride applications are not totally effective for controlling dental caries during orthodontic treatment [21].

The introduced fluoride releasing elastomeric ligatures have been reported to reduce dental biofilm formation and improve enamel remineralization in areas nearby to the brackets base, which are difficult to clean [11]. Benson et al. found that fluoridated elastomers were not effective in the reduction of streptococcal growth after a clinically relevant time [22]. Several studies have investigated the performance of fluoride-releasing elastomers on decreasing both the formation of *S. mutans* colonies or biofilms and the susceptibility for development carious lesions around orthodontic brackets. Generally, the findings of these studies have shown that fluoride-releasing elastomeric rings were not effective for that purpose [11]. Fluorine can inhibit demineralization and promote remineralization of hard dental tissues. But studies indicated that the duration of fluorine release was short-term [5]. Studies found that over half the total fluoride content of fluoride-releasing elastomers stored in vitro was released in the first 24 h, and 90% by the end of the first week [23].

Recently, a product that releases silver ions from silver-zeolite that is incorporated into an elastomer (Orthoshield Safe-T-tie) has been introduced in order to reduce bacterial development around orthodontic appliances. Nevertheless, Kim et al. found there were no significant differences between the antimicrobial effect on the silverized elastomers and the conventional elastomers. This study in vivo suggests that the concentration of released ions was not sufficient to impede bacterial growth [24].

Similarly, Won did not find either *S. mutans* or *Porphyromonas gingivalis* clear zones around silverized elastomers in modified agar disk diffusion test. Silverized elastomers were also ineffective in growth inhibition test when they were in direct contact with these microorganisms. Won speculated that the concentration of the silver ions in the silverized elastomers was insufficient for antimicrobial activity [24,25]. Also, O’Dell reported that silver-releasing elastomeric ligatures not were effective in inhibiting growth of *S. mutans* in vitro [26].

Nevertheless, Bai et al. found these technological modifications of the elastomers are a definite improvement over the regular elastomers with regard to adhesion of *S. Mutans* and *Lactobacilli* [13]. Caccianiga et al. conclude that Orthoshield Safe-T-Tie ligatures reduce gingival inflammation and periodontal pathogens in orthodontic patients. More studies will be necessary [27].

Nanotechnology has been applied to dental materials as an innovative concept for the development of materials with better properties and anti-caries potential. Nanomaterials have great potential to decrease biofilm accumulation, to inhibit the demineralization process and to combat caries-related bacteria [28]. Silver nanoparticles have been synthesized and incorporated into several biomaterials [29]. The use of plants extracts for nanoparticle synthesis may be advantageous over other biological processes, because it drops the elaborate process of maintaining cell cultures and can also be used for large-scale nanoparticle synthesis. Additionally, the green chemistry approach for the synthesis of nanoparticles using plants avoids the generation of toxic byproducts. Among the various known synthesis methods, plant-mediated nanoparticle synthesis is preferred as it is cost-effective, ecofriendly and safe for human therapeutic use [30,31,32]. Phytochemical compounds such as saponins, phenolic compounds, phytosterols and quinines present in plant biomolecules have both preservative and reductive activity [33]. 

Silver nanoparticles (AgNPs) have been synthesized by several methodologies, and they have shown potent antimicrobial properties [29].Many methods have been used for the synthesis of silver nanoparticles, ranging from physical solid-state treatments (including milling, grinding and mechanical alloying techniques) [34], gas-phase synthesis (high-temperature evaporation) [35], laser ablation [36], pyrolisis [37], plasma synthesis to liquid-phase synthesis [38]. The latter includes a variety of methods such as coprecipitation, microemulsifying, microwave irradiation, solvothermal treatments and sol-gel synthesis [39].

However, in most of the methods, hazardous chemicals, low material conversion and high energy requirements are used for the preparation of nanoparticles [40]. Also, employing synthetic stabilizing agents can generate hazardous byproducts, making these methods unsuitable for biological applications [41]. So, there is a need to develop high-yield, low cost, non-toxic and environmentally friendly procedures [42]. In such a situation, the biological approach appears to be very appropriate. Natural materials, like plants, bacteria, fungi, yeast, have been used for the synthesis of silver nanoparticles [40]. The dried flower of *Heterotheca inuloides*, which is called “arnica”, has been used in Mexican traditional medicine to treat inflammatory discomfort [43,44].

In the present study, we synthesized metallic silver nanoparticles using the extract of *Heterotheca inuloides* and evaluated the antibacterial and physical properties of orthodontic elastomeric modules decorated with these silver nanoparticles (AgNPs).

## 2. Results

### 2.1. Characterization of the Silver Nanoparticles Biosynthesized

**Figure 1:**
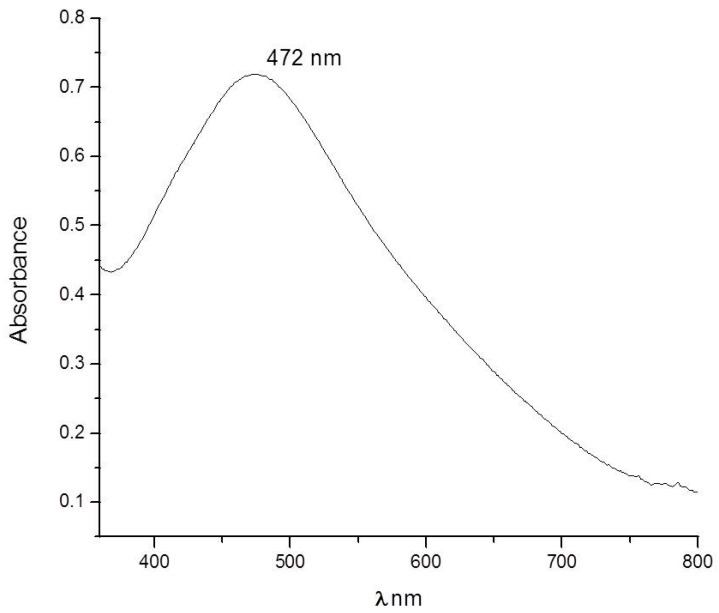
UV-Vis spectra of Ag-NPs obtained with *Heterotheca Inuloides*.

All synthesis parameters were investigated to be able to adequately decorate the elastic modules with silver nanoparticles without being agglomerated but in sufficient quantity to have good antibacterial activity. Pretreated orthodontic elastomeric ligatures were immersed in 8 mL of 1 × 10^−2^ M silver nitrate (AgNO_3_) (Sigma-Aldrich, St. Louis, MO, USA) for 60 min and later 2.5 mL of *Heterotheca inuloides* extract was added to reduce Ag^+^ ions. The synthesis of silver nanoparticles was carried out for 12 h. The bioreduction of AgNO_3_ into AgNPs can be confirmed visually by change in the solution color, from colorless to reddish brown. UV-Vis absorbance of AgNPs shows the characteristic plasmon absorption peak, which was detected at 472 nm (Figure 1). The elastic modules are very transparent originally; after the process of incorporation of silver nanoparticles on the surface, they take a little change of color and appear slightly yellow.

**Figure 2:**
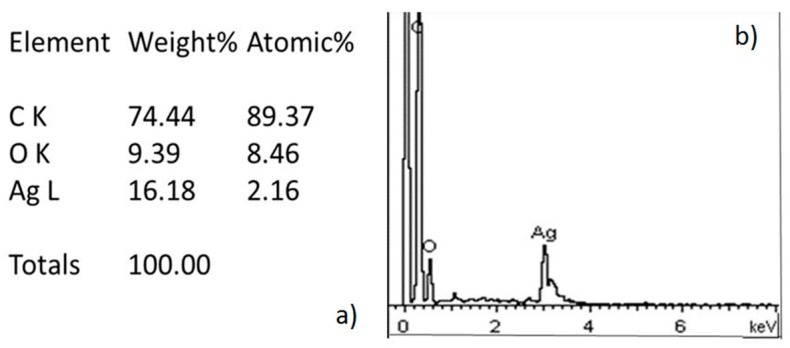
(**a**) Results of the elements ratio obtained for EDS; (**b**) EDS spectrum of Ag-NPs which confirmed the presence of silver.

The energy dispersive spectrometry (EDS) analysis recorded for AgNPs is listed in Figure 2.

**Figure 3:**
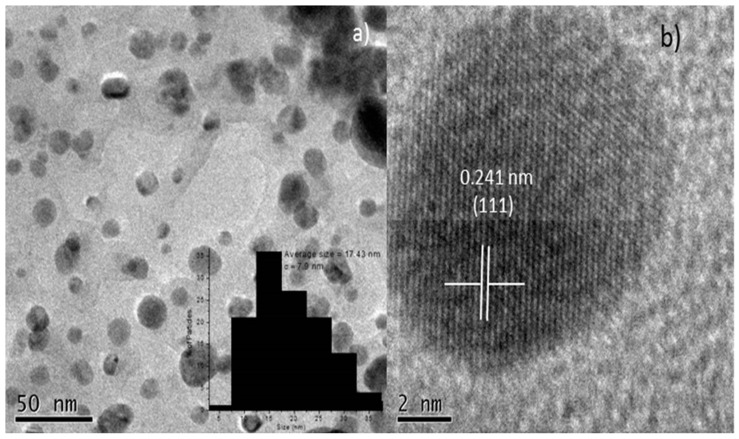
(**a**) TEM image of Ag NPs, inside histogram of size nanoparticles; (**b**) HRTEM image of a Ag-NP.

Figure 3 are TEM images showing that the shape of the Ag-NPs; they tend to be spherical as can be seen in Figure 3a. Inside, the nanoparticles size distribution is very narrow to 17 nm. Figure 3b High Resolution Transmission Electron Microscopy (HRTEM) shows an interplanar distance of 0.241 nm which corresponds to (111) plane of Ag-NPs.

### 2.2. Thermogravimetric Analysis

**Figure 4:**
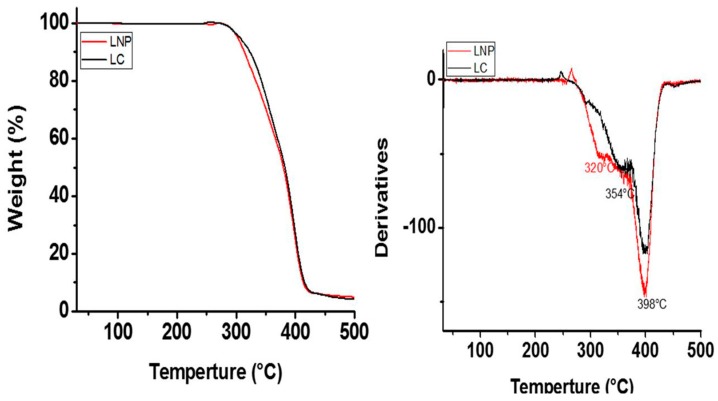
Thermogravimetric analysis curves of the control modules (LC) and modules with Ag-NPs (LNP).

Figure 4 shows Thermogravimetric analysis (TGA) curves of orthodontic elastic modules with and without AgNPs from 30 °C to 500 °C. The TGA curve of orthodontic elastic modules control showed T_5_ at 306 °C, the other stages of degradation temperature were at 354 °C and 398 °C; while in the TGA of orthodontic elastic modules with AgNPs can be appreciated T_5_ at 303 °C, 320 °C and 398 °C. Control orthodontic elastic modules showed lower onset degradation temperature in comparison to orthodontic elastic modules with AgNPs.

### 2.3. Antibacterial Activity

**Figure 5:**
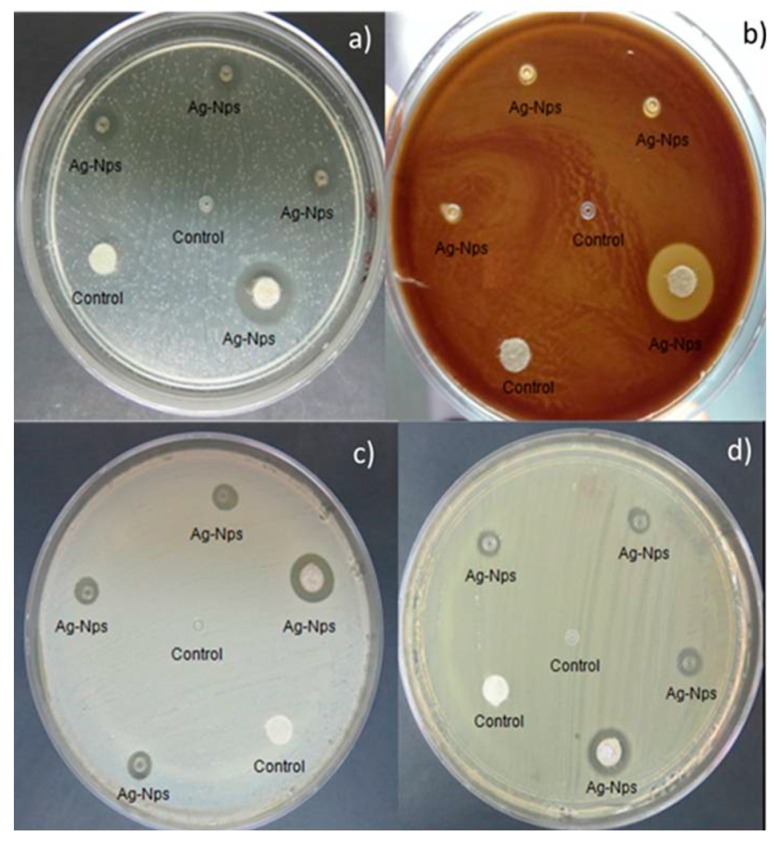
Antibacterial activity of samples in the agar diffusion test. Ag-NPs showed inhibition halos for (**a**) *Streptococcus mutans*; (**b**) *Lactobacillus casei*; (**c**) *Stsphylococcus aureus*; (**d**) *Escherichia coli*.

In addition to evaluating the most common types of bacteria for this research, such as Gram-positive *S. aureus* and Gram-negative *E. coli*, two other common oral cavity microorganisms were evaluated, such as *L. casei* and *S. mutans*, Gram-positive both. The results of the antimicrobial activity are shown in Figure 5. The control sample revealed no activity against all tested microorganisms. Orthodontic elastomeric ligatures containing AgNPs exhibited antibacterial activity against Gram-negative and Gram-positive bacteria. The mean values and standard deviation of the zone of growth inhibition (mm) of orthodontic elastic modules and paper disk are shown in Table 1.

### 2.4. Physical Properties

**Figure 6:**
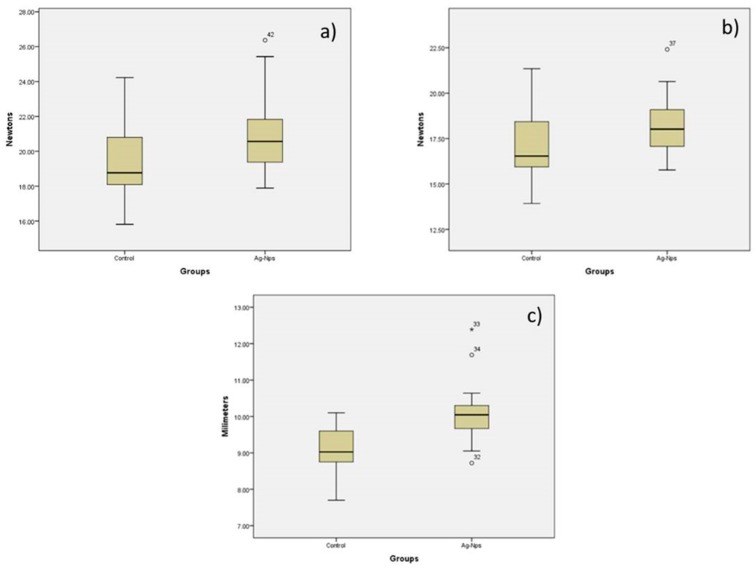
Comparison of orthodontic elastic modules control and orthodontic elastic modules decorated with Ag-NPs for (**a**) Maximum strength; (**b**) Tension; (**c**) Displacement.

The *t*-test revealed there were significant differences between orthodontic elastic modules control and orthodontic elastic modules decorated with AgNPs (*p* < 0.05) (Table 2). Physical properties (maximum strength, tension and displacement) of orthodontic elastic modules with AgNPs increased with respect to control group (Figure 6).

## 3. Discussion

The reduction of silver ions was considered to occur due to the phenolic components present in the extract of *Heterotheca inuloides* [45]. Further studies are required to establish the mechanism of formation and stabilization of nanoparticles.

The biosynthesis of AgNPs was initially observed by the color change from colorless to reddish brown. The color change is due to the excitation of surface plasmon resonance vibration in AgNPs. Similar results were observed with various plants like studied by Sudhakar et al. and Joy Prabu et al. [46,47]. Generally, the characteristic part of the surface plasmon band of AgNPs falls within the wavelength range of 350–500 nm [48]. The appearance of surface plasmon peaks around 472 nm and confirms the formation of AgNPs. The kinetics of formation of silver nanoparticles by bioreduction usually occurs at 6 h and particularly with *H. inuluoides*, we find that if we leave in contact the elastic modules with silver nanoparticles for 12 h, we achieve a higher concentration. This reaches 16% of the weight of silver nanoparticles without agglomeration occurred; this assures us a high rate of antibacterial effectiveness.

The elastomeric ligatures were made of polyurethane, which are thermosetting polymers, that have a -(NH)-(C=O)-O- structural unit and are formed by step reaction (condensation) polymerization. The manufacture of polyurethane elastomers involves several stages. These polymers have short rigid portions (the aromatic rings and the urea) joined by short flexible hinges (the diamine linker and the CH_2_ group between the aromatic ring) and long very flexible portions (the polyether) whose length can be adjusted [49]. These functional groups provide links for binding AgNPs.

Thermogravimetric analysis curves show differences in the thermal stability of the control modules and modules with AgNPs. The result of this involvement is mainly due to exposure to pretreatment with NaOH that could generate a degree of surface hydrolysis of the urethane groups. However, these differences did not affect the antibacterial properties and the physical properties of AgNPs; this is demonstrated by the studies carried out on the elastic modules after the treatments with isopropyl alcohol and with sodium hydroxide, and also with the incorporation of silver nanoparticles. In contrast, the physical properties increased in a small proportion; however, further studies should be carried out to evaluate in detail the stability and all physical and mechanical properties.

Silver has superior antibacterial activity compared to other metals; it has a strong cytotoxic effect on a broad range of microorganism in metallic and ionic forms. Several studies have evaluated the cytotoxicity of silver nanoparticles on fungi, protozoa, a number of viruses, and Gram-negative and Gram-positive bacterias such as *Streptococcus mutans, Lactobacillus* sp., *Escherichia coli* and *Staphylococcus aureus*, confirming the antibacterial and bactericidal properties of silver nanoparticles [50,51,52,53]. Hernández-Sierra et al. indicated that AgNPs inhibits the growth of *S. mutans* at lower concentrations compared to Zn-Nps and Au-Nps and thus it may be more effective against dental caries [54]. Our results show that orthodontic elastic modules decorated with silver nanoparticles inhibited not only the bacteria on the materials surfaces, also the bacteria away from the material in the culture medium against *S. mutans, L. casei, S. aureus* and *E. coli*. This indicates the potential ability of this materials to combat incidence of enamel decalcification in orthodontic patients because there showed significant reduction in *S. mutans* and *L. casei.*

The mechanism of antibacterial activity is not very well-known; possibly the AgNPs inhibits the enzymes of the cell respiratory cycle and damages the deoxyribonucleic acid (DNA) synthesis, leading to cell death [54,55]. In the present study, the Ag salt was reduced to AgNPs in situ, avoiding the need for prefabricated nanoparticles to be mixed with the polymer, which could cause agglomeration. The high surface area of AgNPs provided potent antibacterial effect with better physical properties, except, changes in color, from clear to light yellow as a result of incorporation of AgNPs in the orthodontic elastic modules. In all probability, the colour appearance of the tooth will not be affected by the addition of Ag-Nps, like shown the study realized by Argueta-Figueroa [56], this is because the Ag-Nps were synthesized in situ on the modules and the existing Van Der Walls interactions between positively charged nanoparticles have strong attraction to the support (modules). In addition, these modules are changed every month during the treatment review. Direct comparison of these results with others studies is difficult because there are no similar published studies.

Ag-Nps have also been applied in several areas of dentistry, as endodontics [57,58], dental prostheses [59,60], implantology [61,62], restorative dentistry [63,64], and orthodontic adhesives [65,66]. Nanomaterials provide superior antimicrobial activity and display comparable physical properties when compared with conventional materials—this is probably due to the small size and high surface area of the nanoparticles [28,67]. Nevertheless, the oral environment is dynamic, with constant changes in temperature, pH, and the volume of fluids washing over the modules; a further complication could be differences in diet, salivary flow rates, and oral-hygiene regimens [23]. This study was performed in vitro and the physiological conditions of in vivo studies may differ [68]. More precise methods are necessary to simulate more precisely the dynamic relationship between wire, bracket, and ligature during tooth movement. Further in vivo studies should be performed to determine the long-term performance of orthodontic material using nanotechnology.

Silver is known to have low toxicity and good biocompatibility with human cells [69]. However, further specific studies are needed to determine its cytotoxicity when AgNps are attached to orthodontic elastic modules.

## 4. Materials and Methods

### 4.1. Experimental and In Vitro Study

#### 4.1.1. Pre-Treatment of Orthodontic Elastic Ligatures

Orthodontic elastomeric ligatures (Mini Stix ligature ties non-coated, TP Orthodontics, LaPorte, IN, USA) were immersed in isopropyl alcohol and cleaned in an ultrasonic cleaner (Branson 1510R-DTH, Branson Ultrasonics, Danbury, CT, USA) for 30 min, rinsed with deionized water, and added NaOH 10%. After that, orthodontic elastomeric ligatures were put in an ultrasonic cleaner one more time for 30 min and then rinsed several times with deionized water.

#### 4.1.2. Preparation of the *Heterotheca Inuloides* Extract

1 g of *Heterotheca inuloides* from Anahuac Mexican teas (99.90% of purity) was boiled for 5 min in 100 mL of deionized water and then filtered. The aqueous extract was used as the reducing agent for synthesis of silver nanoparticles [70].

#### 4.1.3. In Situ Synthesis of AgNPs in Orthodontic Elastic Ligatures

Pretreated orthodontic elastomeric ligatures were immersed in 8 mL of 1 × 10^−2^ M silver nitrate (AgNO_3_) (Sigma-Aldrich, St. Louis, MO, USA) for 60 min and later 2.5 mL of *Heterotheca inuloides* extract was added to reduce Ag^+^ ions. The synthesis of silver nanoparticles was carried out for 12 h into the darkness (to minimize the photoactivation of silver nitrate). Later, orthodontic elastomeric ligatures were removed from the solution and allowed to dry at room temperature during 8 h.

### 4.2. Characterization of AgNPs

Reduction of Ag^+^ ions was assessed by measuring the UV-Vis spectrum of 1 mL aliquots of the sample in a quartz cell as described forward. UV-Vis spectral analysis for AgNPs was carried in a Cary 5000 UV-Vis Spectrophotometer. Measurements were performed in an interval between 200 and 800 nm range operated at a resolution of 1 nm. 

Synthesized AgNPs were characterized by scanning electron microscopy (SEM) energy dispersive spectrometry (EDS) (JEOL, JSM-6510LV, Tokyo, Japan) at 20 kV of acceleration and using secondary electrons and transmission electron microscopy (TEM) was carried on in a JEOL-2100 microscope (Tokyo, Japan) at 200 kV of acceleration in the bright field mode. In order to prepare the samples from TEM the specimens were sonicated during 3 h to detach the nanoparticles from the orthodontic elastomeric ligature.

### 4.3. Characterization of Orthodontic Elastic Ligatures Decorated with AgNPs

#### 4.3.1. Thermogravimetric Analysis 

Thermal stability of the conventional and orthodontic elastic modules with AgNPs examined by thermogravimetric analyses (TGAs) using SDT (Q600 model). The weight chance of each sample was evaluated by TGAs at a heating rate of 10 °C/min to 600° in a nitrogen atmosphere (flow of 100 mL/min).

#### 4.3.2. Antibacterial Activity

The in vitro antibacterial activity of the samples was determined using a direct contact test with agar diffusion technique according the Clinical and Laboratory Standards Institute (CLSI) [71]. Mueller-Hinton agar (MHA) (BD Bioxon, Spark, MD USA) were prepared and inoculated with bacterial culture. Mueller-Hinton agar with 5% sheep blood was necessary to testing of *L. casei.*

Bacterial strains used in this study were obtained from the culture collection of the Biochemistry Laboratory of the School of Dentistry, National Autonomous University of Mexico (UNAM). Strains used are endemic to the region from central Mexico, and each one was characterized by cultural and biochemical test [72].

Antibacterial activity of AgNPs was investigated against a panel of clinically relevant microorganisms, representative for Gram-positive and Gram-negative bacteria commonly used as standards: *S. aureus*, *E. coli*, *S. mutans* and *L. casei*.

The culture was adjusted with sterile saline to achieve a turbidity equivalent to a 0.5 McFarland standard or 10^8^ CFU/mL. The agar plates were inoculated from the standardized cultures of the test microorganisms using a sterile cotton swab and then spread as uniformly as possible throughout the entire media. Three orthodontic elastomeric ligatures with AgNPs, one orthodontic elastomeric ligature control, one disk made of filter paper was impregnated with ten µL of AgNPs concentration of and one disk control were firmly placed on agar plates. Inoculated agar plates were incubated at 37 °C for 24 h. Agar plates with *S. mutans* and *L. casei* were incubated in anaerobic jar. Antibacterial activity was evaluated by measuring the diameter of the inhibition zone (mm) on the surface of the plates, and the results were reported as mean ± standard deviation. The antimicrobial activity was assessed using procedures from the Clinical and Laboratory Standards Institute [52].

#### 4.3.3. Mechanical Properties

Mechanical properties (maximum strength, tension and displacement) of orthodontic elastic ligatures decorated with AgNPs and conventional ligature were tested by universal testing machine (Autograph AGS-X, Shimadzu, Kyoto, Japan). Using a U-shaped hook adapted to the machine, elastomeric ligatures were stretched until they were broken. This was carried out with a crosshead speed of 100 mm/min. As each elastomer was stretched, force (newtons) and extension (mm) were measured and recorded.

The maximum force was operationally defined as the ability to move the maximum weight for a single repetition; tension as the effect of applying a force on a shape increasing its elongation; and the displacement was the change in position.

## 5. Conclusions

We have demonstrated that silver nanoparticle biosynthesis by *Heterotheca inuloides* promises an ecofriendly, non-toxic, simple and economical pathway to synthesize AgNPs with a controlled average size of 17 nm and stable. UV-visible spectroscopy showed peaks in the range of 472 nm confirming the formation of AgNPs. Orthodontic elastic modules decorated with AgNPs can inhibit the growth of three important Gram-positive microorganisms commonly found in oral cavities: *S. mutans*, *L. casei* and *S. aureus* as well as Gram-negative bacteria like *E. coli*, demonstrated that the composite possesses broad spectrum antibacterial activity. Orthodontic elastic modules decorated with AgNPs demonstrated higher physical properties such as maximum strength, tension and displacement compared to conventional modules. The results suggest the potential of the composite to combat dental plaque and therefore decrease the incidence of dental enamel demineralization, ensuring its performance in patients with orthodontic treatment.

## Figures and Tables

**Table 1:** Inhibition zone (mm).

Microorganism	Mean and Standard Deviation	
	Ligature with AgNPs	Paper Disk with AgNPs
*S. mutans*	2.0 ± 0.12 mm	4.0 ± 0.16 mm
*L. casei*	1.0 ± 0.21 mm	5.0 ± 0.27 mm
*S. aureus*	2.0 ± 0.18 mm	3.0 ± 0.22 mm
*E. coli*	1.5 ± 0.12 mm	2.0 ± 0.15 mm

**Table 2:** Physical properties in orthodontic elastic modules.

Physical Properties	Mean		Range		* *p*
	Control	AgNPs	Control	AgNPs	
Maximum strength	19.4897	20.8370	15.81–24.23	17.89–26.37	0.012
Tension	17.2320	18.1847	13.93–21.35	15.77–22.41	0.033
Displacement	9.0667	10.0733	7.70–10.10	8.72–12.39	0.001

* *p* value ≤ 0.05 according to *t* test.
